# Development of Low-Shrink Epoxy Putty to Solve Appearance-Quality Defects of Carbon-Fiber-Reinforced Plastic Automotive Exterior Parts

**DOI:** 10.3390/ma14216419

**Published:** 2021-10-26

**Authors:** Manseok Yoon, Kwangsik Yoo, Bongkuk Seo, Seung Hwan Ko, Choong-Sun Lim

**Affiliations:** 1 School of Mechanical and Aerospace Engineering, Seoul National University, Seoul 08826, Korea; alex76@snu.ac.kr (M.Y.); maxko@snu.ac.kr (S.H.K.); 2 Kukdo Chemical, Seoul 08588, Korea; yksyjk81@naver.com; 3 Research Center for Advanced Specialty Chemicals, Korea Research Institute of Chemical Technology, 45, Jongga-ro, Yugok-dong, Jung-gu, Ulsan 44412, Korea; bksea@krict.re.kr

**Keywords:** composites, putty, epoxy, unsaturated polyester resin

## Abstract

In this study, epoxy putties with novel compositions were developed for the filling of structural voids in carbon-fiber-reinforced plastics (CFRPs), which are used to fabricate automotive parts. Two constituent solutions—one consisting of epoxy resins and the other consisting of a hardener—were formulated, mixed, and then coated on CFRP surfaces, followed by curing. The surfaces were then evaluated to determine the shrinkage rates (calculated based on the liquid densities and cured mixtures), adhesion properties (determined by a peel test), and color differences (measured with a colorimeter) of the synthesized putties. The last two properties were compared with those of the commercially available putties to ascertain the thermal resistance of the developed putties. The results indicated that the synthesized epoxy putties were more strongly adhesive and exhibited less difference in color. Furthermore, after thermal impact, both the adhesive properties and color stabilities of the synthesized epoxy putties were found to be superior to those of the commercial putty.

## 1. Introduction

Carbon-fiber-reinforced plastics (CFRPs) are widely used in the aircraft, automotive, and construction industries owing to their low weight and high strength [1,2,3,4,5,6,7,8,9,10]. However, during the compression molding process involved in their manufacturing, inhomogeneous heat transfer and resin flow frequently lead to the formation of structural voids [11]. These voids can deteriorate both the surface quality and mechanical properties [12,13,14,15,16,17,18,19]. Although the formation of voids during the resin-transfer molding process can be reduced by controlling the curing process, the rate of resin impregnation, and the direction of resin flow [20,21,22,23,24], the complete elimination of such voids is crucial for aesthetic purposes and for maintaining the quality of the coating. Additionally, because voids always exist in CFRPs, the minimization of void formation during the preparation of CFRPs is critical [25]. The parts of automobiles are composed of inner plates and outer plates. For the fabrication of the inner plates of composite materials, materials with a void content above 2% are rejected, as they often do not provide the required structural performance [26]. However, because the requirements for the mechanical properties of the outer plate materials are not as stringent, a higher void content is acceptable, and thus putty is often used to fill the surface defects.

Surface voids are usually treated by the conventional painting method. However, this is not always feasible due to the considerable time and cost required to cover all of the voids. In the case of iron or aluminum products, the defects are painted only after the voids are filled with putty. However, CFRPs contain voids throughout their structures; thus, if the number of voids increases, more appearance defects may manifest themselves after painting the entire product surface, compared to painting just a specific part. Therefore, contrary to the traditional puttying method, which is used only on specific parts of the metal surfaces, in the case of a CFRP, the putty is sprayed over its entire surface. In addition, when CFRPs are used to fabricate the exterior parts of vehicles, transparent paint is applied to them to enhance their visual appeal for marketing purposes. Thus, the putty must be transparent as well so that the carbon-fiber pattern can be seen with the naked eye. Therefore, transparent putty is generally applied over the voids before the coating process [27]. Transparent putty based on unsaturated polyester resin (UPR) is generally used as a filler. However, its poor adhesion and high shrinkage limit its applicability. In particular, when the shrinkage rate is high, even if all the voids are completely filled with putty, it shrinks at some point after the completion of painting and curing, leading to the formation of pinhole defects. Therefore, to reduce the rate of defect formation in CFRPs that are used in automobile exterior panels, the putty that is used must be transparent as well as exhibit a high penetration rate, low shrinkage, and high adhesion.

In this study, a novel putty based on a transparent epoxy resin with fortified adhesion to CFRPs was developed in order to overcome the disadvantages of UPR putty [28,29]. In addition, the physical properties of CFRPs that were treated with traditional unsaturated polyester putty were analyzed using the cross-cut adhesion method and by comparing the color of the samples before and after exposure to high temperatures. Two mixtures—one composed of resin and the other of hardener—were prepared for the experiments. Diglycidyl ether of bisphenol A (DGEBA) was chosen as the resin for reducing the shrinkage rate. For the hardener, four different compositions of triethylene tetramine (TETA), diethylene tetramine (DETA), bisphenol A (BPA), and isophorone diamine (IPDA) were studied. The newly developed putty systems and the UPR putty were tested using the same methods, and the corresponding results were compared.

## 2. Materials and Methods

### 2.1. Materials

Carbon clear putty 3K (Gelson, Milan, Italy) was purchased and used without further treatment. Prepreg carbon fiber (SK Chemicals, Seongnam, Republic of Korea) was used as the symmetrically laminated reinforcement. The outermost layers (1st and 4th plies) were woven with Toray T700 carbon fibers (3K; fiber areal weight (FAW): 200 g/m^2^) with an orientation of 0°/90°. In addition, the inner layers (2nd and 3rd plies) were woven with Zoltek (50K; FAW: 240 g/m^2^) non-crimp fabrics with an orientation of +45°/−45°. The carbon fabric prepreg details are shown in Table 1.

DGEBA was obtained from Kukdo Chemical (Seoul, Korea), and TETA was purchased from Huntsman (Woodlands, TX, USA). IPDA and DETA were purchased from Merck (Darmstadt, Germany). NRB DP 2411- and NRB DC 2411-type primers were obtained from NoRoo Bee Chemicals (Seoul, Korea). All of the chemicals were used without further purification.

### 2.2. Preparation of CFRP Test Specimens

**Figure 1:**
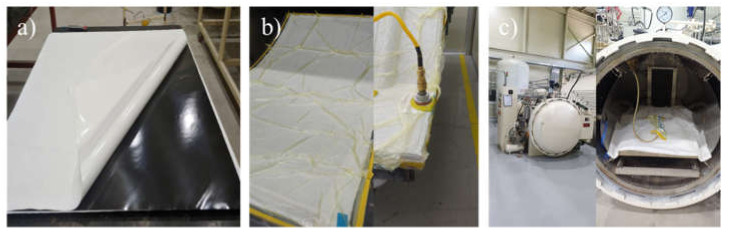
CFRP-preparation process: (**a**) material cutting and lamination, (**b**) vacuum processing, and (**c**) autoclave curing.

**Figure 2:**
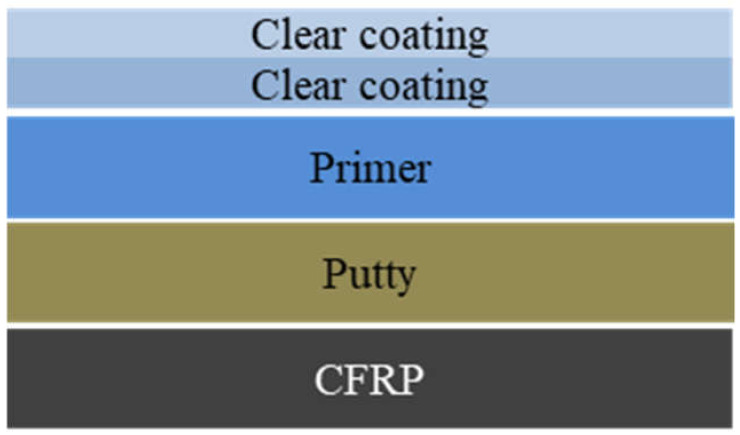
Coating process after putty treatment.

The prepreg carbon fibers were cured for 5 h in an autoclave that was maintained at a pressure of 3 bar. The curing temperature was steadily increased to 140 °C, at a rate of 2 °C/min. After this curing process, the oven was allowed to cool naturally to room temperature (Figure 1). The laminates were first cleaned with acetone and then sanded with sandpaper. Thereafter, the putty was sprayed on the laminates under an air pressure of 2–3 bar until it formed a 30–50 μm-thick film, followed by curing of the sample at 60 °C for 1 h. The NRB DP 2411 primer was then sprayed on the sample with an air spray gun until it formed a 50–60 μm-thick film, followed by sanding with sandpaper. Then, the sample was again cured at 60 °C for 1 h. For the clear-coating process, the laminates, cleaned with acetone, were sprayed with the NRB DC 2411 primer until they formed a 50–60 μm thick film and were subsequently cured at 60 °C for 1 h. Finally, the sample was again sprayed with 50–60 μm of NRB DC 2411 and cured at 75 °C for 2 h, as shown in Figure 2.

### 2.3. Preparation of Epoxy Compositions and Curing

Four different epoxy putties were prepared by mixing DGEBA with curing agents of four distinct compositions, as shown in Table 2.

The components were mixed and stirred for 2 h by a mechanical stirrer operating at 1000 rpm and then sprayed onto the prepared CFRPs with a spray gun.

### 2.4. Characterization and Analysis

The thickness of the sprayed putty was measured using an optical microscope (DM6000 M, Leica, Wetzlar, Germany). The cross-cut adhesion test was conducted on the putty in accordance with the ISO 2409 standards: First, the test sample and a cutting guide were placed on a horizontal surface and a coated surface, respectively. Next, the coating was penetrated with a cutting knife, which drew a line inclined at 30° with respect to the material. This process was repeated to draw 10 such lines at 2 mm intervals along the coated surface, after which 10 additional vertical lines were drawn to form a total of 100 intersections. Finally, adhesive tape was attached to the surface and pulled off within 0.5 s in the 90° direction, and the detached surface was observed and rated based on the scale provided by the test method. The results of the experiments were classified from class 0 to class 5.

The color difference (∆E* or *dE*) was measured using a colorimeter (Chroma Meter CR-400, Konica Minolta, INC., Tokyo, Japan), by comparing the L*a*b* coordinates between the reference and the thermally treated samples.

The heat resistance of the test sample was evaluated by leaving the samples in an oven at 80 °C for 300 h, after which they were cooled to room temperature for 1 h.

The results of the adhesion tests and color-difference measurements were compared with those of the UPR reference samples. The structure of the UPR putty was analyzed by Fourier transform infrared (FTIR) spectroscopy (670 IR, VARIAN, Santa Clare, CA, USA) and pyrolysis gas chromatography mass spectrometry (PyGCMS, Focus GC/ISQ, THERMO, Waltham, MI, USA). The gel time was measured with a Giken GT-D (Eucaly, Kawakuchi, Japan) by placing the resin on a hot plate equipped with a wire stirrer at 70 °C, whereas the viscosity was determined using a viscometer (1/23 CAP 2000+H, Brookfield, Middleboro, MA, USA) by following the ASTM D2196 method. The shrinkage rate was calculated by using Equation (1) and the ISO 3521 method.

(1) $$\mathrm{Shrinkage}\;\left(\%\right)=\frac{\left\{\left(\frac{1}{\mathrm{density}\;\mathrm{of}\;\mathrm{liquid}\;\mathrm{mixture}}\right)-\left(\frac{1}{\mathrm{density}\;\mathrm{of}\;\mathrm{cured}\;\mathrm{specimen}}\right)\right\}}{\left(\frac{1}{\mathrm{density}\;\mathrm{of}\;\mathrm{liquid}\;\mathrm{mixture}}\right)}\times 100$$

The curing behavior of the DGEBA–IPDA compositions was monitored using differential scanning calorimetry (DSC; Q2000, TA Instruments, New Castle, DE, USA) over the operating temperature range from 25 to 250 °C and at a heating rate of 10 °C/min to obtain ∆*H*.

## 3. Results and Discussion

**Figure 3:**
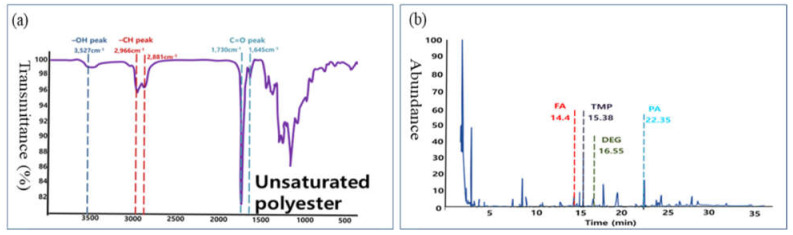
Analysis of UPR putty using (**a**) FTIR spectroscopy and (**b**) PyGCMS (FA: fatty acids; TMP: trimethylolpropane; PA: phthalic anhydride).

Commercial UPR putty was analyzed by using FTIR and PyGCMS. The corresponding results are shown in Figure 3. The FTIR spectrum (Figure 3a) shows the existence of an ester C=O in the UPR (peak at 1730 cm^−1^), whereas the PyGCMS spectrum shows the presence of monomers of fatty acids, trimethylolpropane, diethylene glycol, and phthalic anhydride in the UPR (Figure 3b).

**Figure 4:**
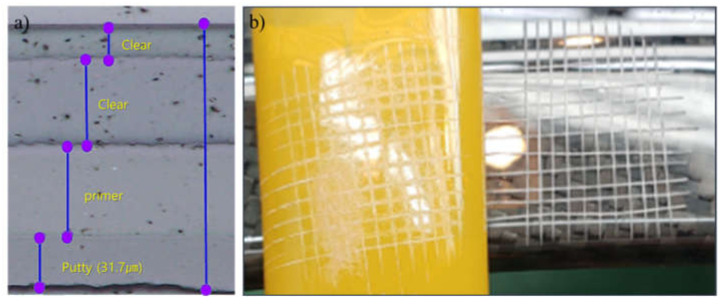
(**a**) Coating layers under an optical microscope and (**b**) an example of the adhesion test.

The observation of the coated layers under a microscope revealed that each layer was uniform and well-arranged (Figure 4a). The adhesion property (Figure 4b) of the UPR-treated laminated samples exhibited degradation with an increase in the putty thickness; that is, it changed from class 0 (the edges of the cuts are clean) for the 52.0 μm thick putty to class 0.5 (i.e., between class 0 and class 1, indicating that less than 5% of the cross-cut area was detached) for the 62.6 μm thick putty, as depicted in Table 3. Furthermore, the color differences of the heated samples increased with increasing putty thickness, exhibiting the maximum difference of 2.33 at a thickness of 188.6 μm. The weak adhesion strength and high color difference of the commercial putty after heat treatment demonstrate the requirement for a new thermostable putty system.

The data presented in Table 4 indicate that the four different epoxy-based putties had sufficient flow properties (with viscosities < 2000 cP) to fill the mold during the CFRP-preparation process. Furthermore, compared to the UPR (shrinkage rate: 7.1% at 70 °C), all four experimental samples exhibited lower shrinkage rates at 70 °C (2.8–4.2%).

**Figure 5:**
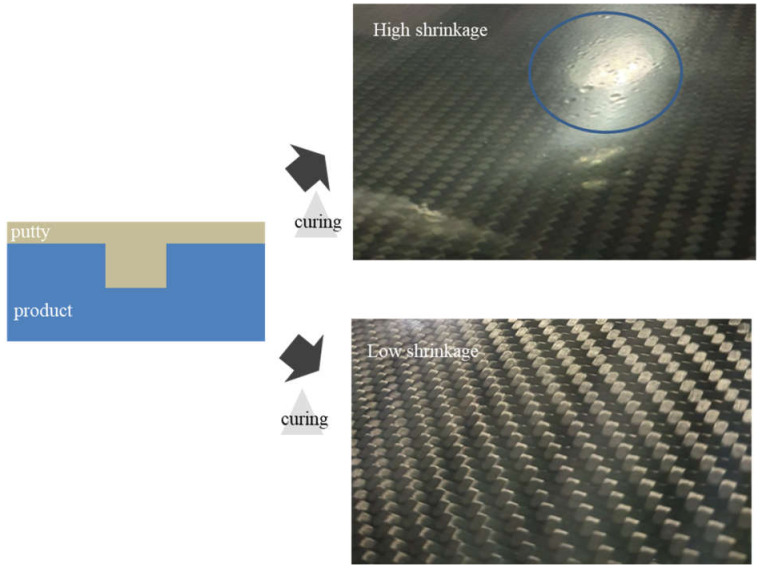
Low shrinkage rate of putty can reduce the number of pinholes after curing.

**Figure 6:**
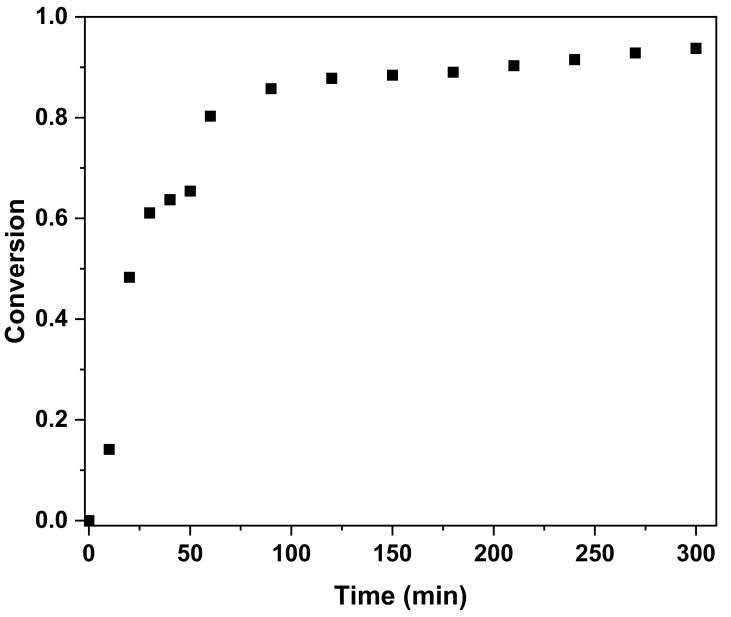
Cure conversion of the DGEBA–IPDA system heated at 60 °C for 3 h and then at 75 °C for 2 h.

Table 4 indicates that although H-4 showed an excellent shrinkage rate of 2.8%, which was much lower than those of the other samples, it exhibited a relatively long gel time of 10.25 min and a low viscosity, both of which lengthen the time during which the resin would be wet in the carbon fiber. Specifically, a low shrinkage rate of the putty can curtail the generation of pinholes after curing, as shown in Figure 5. Considering the gel time, low viscosity, and shrinkage rate, sample H-4 was chosen for further analysis. First, the curing behavior of H-4 was studied via DSC measurements in order to calculate the degree of conversion (α) as a function of time using Equation (2) (Figure 6) [30].

*α*(*t*) = ∆*H_t_*/∆*H*_0_, (2)

where *t* is curing time, *α* is the fractional conversion by curing, ∆*H_t_* is the amount of heat released for time *t*, and *∆H*_0_ is the total reaction heat in the system.

Figure 5 clearly shows that the degree of conversion of H-4 converges to a point over 0.9 after 210 min.

The changes in the physical properties of the H-4 sample and UPR epoxy putty after thermal exposure were also observed by conducting adhesion and color-difference tests (Table 5). The adhesion rating of a 64.9 μm-thick film of the H-4 sample was found to be M-1.0, whereas that of the UPR putty with a comparable thickness of 62.6 μm was found to be M-1.5. Additionally, the H-4 sample after heat exposure showed a low color difference of 0.67–0.89 dE when the coating thickness was in the range of 47.8–78.9 μm, whereas the UPR-treated CFRPs, after exposure to heat, showed more discernible color differences ranging from 1.1 to 1.63 dE for coating thicknesses from 31.7 to 62.6 μm. Both of these results suggest that when compared to the CFRPs that were treated with commercial UPR putty, those treated with the epoxy putty that was synthesized in this study are much more strongly adhesive and exhibit smaller color differences against thermal shock.

## 4. Conclusions

In this study, novel epoxy compositions were formulated as putties and applied to CFRP surface defects, which were then coated with primers. The developed epoxy putty demonstrated lower shrinkage rates than the commercially available UPR putty, as well as a stronger adhesion and higher heat resistance according to the results of the cross-cut adhesion and color-difference tests. Specifically, the H-4 composition displayed a 60.6% lower shrinkage rate than the UPR putty. Furthermore, it showed an adhesion rating of M-1.0 at a thickness of 64 µm, whereas the UPR putty exhibited an adhesion of M-1.5 at a comparable thickness of 62.6 µm. The sample H-4 also exhibited a lower color difference of 0.75 dE, compared to the UPR putty, which showed a more discernible color difference of 1.63 dE for approximately the same thickness.

## Figures and Tables

**Table 1:** Carbon fabric prepreg information.

	FAW ^1^ (g/m^2^)	Angle (°)	Thickness (mm)	Fiber
1st ply (WSN 03KT)	200	0/90	0.224	Toray T700, 3K
2nd ply (WSN 50KMA)	240	±45	0.267	Zoltek, 50K
3rd ply (WSN 50KMB)	240	±45	0.267	Zoltek, 50K
4th ply (WSN 03KT)	200	0/90	0.224	Toray T700, 3K

^1^ FAW, fiber areal weight.

**Table 2:** Compositions and viscosities of the developed and commercial putties.

Sample Name	Resin	Hardener	Resin-to-Hardener Mixing Ratio (g)
H-1	DGEBA	TETA	100:14
H-2		DETA:BPA (7:3)	100:18
H-3		IPDA:TETA (7:3)	100:19
H-4		IPDA	100:24
Commercial UPR putty			100:2.5

**Table 3:** Adhesion property and color difference of UPR-putty-treated samples.

Thickness of Putty (μm)	Adhesion Property		Color Difference (in *dE*)
	UPR	UPR (after Curing)	UPR (after Curing)
31.7	Class 0	Class 0	1.10
40.5	Class 0	Class 0	0.97
52.0	Class 0	Class 0.5	1.54
62.6	Class 0.5	Class 1.5	1.63
188.6	Class 1.5	Class 4	2.33

**Table 4:** Gel time, viscosity, and shrinkage rate of the epoxy putties.

Sample Name	Gel Time at 70 °C (min)	Mixed Viscosity (cP)	Shrinkage at 70 °C (%)
H-1	3.10	1600	4.2
H-2	1.5	1500	4.1
H-3	6.10	1300	3.4
H-4	10.25	1000	2.8
Commercial UPR putty	3.35	255	7.1

**Table 5:** Adhesion property and color differences of the epoxy-putty-treated samples.

Thickness of Putty (μm)	Adhesion Property after Curing	Color Difference after Curing (dE)
47.8	Class 0	0.67
54.0	Class 0	0.71
64.0	Class 0	0.75
78.9	Class 0.5	0.89
87.2	Class 00.5	0.91

## Data Availability

The datasets generated during and/or analyzed during the current study are available from the corresponding author upon reasonable request.
